# Implementation of a learning healthcare system for sickle cell disease

**DOI:** 10.1093/jamiaopen/ooaa024

**Published:** 2020-10-23

**Authors:** Robin Miller, Erin Coyne, Erin L Crowgey, Dan Eckrich, Jeffrey C Myers, Raymond Villanueva, Jean Wadman, Sidnie Jacobs-Allen, Renee Gresh, Samuel L Volchenboum, E Anders Kolb

**Affiliations:** o1 Nemours Sickle Cell Center of Biomedical Research Excellence (COBRE), Nemours Alfred I. duPont Hospital for Children, Wilmington, Delaware, USA; o2 Nemours Center for Cancer and Blood Disorders, Nemours Alfred I. duPont Hospital for Children, Wilmington, Delaware, USA; o3 Department of Pediatrics, Nemours Alfred I. duPont Hospital for Children, Wilmington, Delaware, USA; o4 Information Systems Clinical Applications, Nemours Alfred I. duPont Hospital for Children, Wilmington, Delaware, USA; o5 Center for Research Informatics, University of Chicago, Chicago, Illinois, USA

**Keywords:** electronic healthcare records, knowledgebase, learning healthcare system, sickle cell disease, clinical informatics

## Abstract

**Objective:**

Using sickle cell disease (SCD) as a model, the objective of this study was to create a comprehensive learning healthcare system to support disease management and research. A multidisciplinary team developed a SCD clinical data dictionary to standardize bedside data entry and inform a scalable environment capable of converting complex electronic healthcare records (EHRs) into knowledge accessible in real time.

**Materials and Methods:**

Clinicians expert in SCD care developed a data dictionary to describe important SCD-associated health maintenance and adverse events. The SCD data dictionary was deployed in the EHR using EPIC SmartForms, an efficient bedside data entry tool. Additional data elements were extracted from the EHR database (Clarity) using Pentaho Data Integration and stored in a data analytics database (SQL). A custom application, the Sickle Cell Knowledgebase, was developed to improve data analysis and visualization. Utilization, accuracy, and completeness of data entry were assessed.

**Results:**

The SCD Knowledgebase facilitates generation of patient-level and aggregate data visualization, driving the translation of data into knowledge that can impact care. A single patient can be selected to monitor health maintenance, comorbidities, adverse event frequency and severity, and medication dosing/adherence.

**Conclusions:**

Disease-specific data dictionaries used at the bedside will ultimately increase the meaningful use of EHR datasets to drive consistent clinical data entry, improve data accuracy, and support analytics that will facilitate quality improvement and research.

## OBJECTIVE

The research aims of this project are (1) to improve the consistency of bedside data entry through implementation of a data dictionary, (2) to improve the real-time extraction and visualization of complex electronic healthcare record (EHR) data, and (3) to use sickle cell disease (SCD) as a model demonstrating methods by which EHR data can be displayed via a custom application to enable easy access to information necessary to inform adherence to clinical care guidelines and outcome monitoring in patients with chronic illnesses.

## BACKGROUND AND SIGNFICANCE

The development of efficient compilation and visualization strategies for EHR data is essential for identifying population and individual trends within a disease cohort. This concept demands a purposeful approach to sustainable data collection^1^ to address both patient care and research priorities. It is common for data collection methods in current clinical workflows to include poorly structured data elements, data inaccuracies, and incomplete and inefficient data capture. These issues represent major limitations in the application of EHR-based research and quality improvement efforts.^2^

Advancement toward a learning healthcare system (LHS) focused on evidence-based, value-driven, and patient-centered care is difficult to implement without a robust EHR infrastructure^2^ and relevant data governance policies and procedures. Multidisciplinary and systematic efforts to address inefficiencies in data entry are needed to overcome inadequacies in warehoused data. Informed data engineering, cleaning, and analytics will provide immediate and positive feedback to alter clinical workflows and data entry. This cyclic process can ultimately inspire consistent and sustainable changes, permitting consistent granular data capture. Informed and accurate data entry at the point of care is incentivized through real-time improvements in clinical care and efficiency.^1^

Collectively, a well-integrated system for data entry, extraction, integration, and visualization is essential for effective monitoring and management of chronic illnesses. Improving the availability of granular data elements can facilitate complex and meaningful data queries to define disease phenotypes, positively influence patient care and facilitate research. Using SCD as a model, we have created a standardized data collection workflow that enables granular data entry, coupled with a novel and innovative data visualization platform, in order to improve the care of patients with SCD at both the individual and population levels.

SCD refers to a group of inherited chronic hemolytic anemias caused by a single mutation in the beta globin gene. Disease severity is highly variable and can include wide-ranging acute and chronic complications such as painful vaso-occlusive crises (VOCs), acute chest syndrome (ACS), stroke, splenic sequestration, and other organ damage and/or failure.^3–5^ SCD is a chronic disorder with a wide range of subphenotypes and complications (referred herein as SCD-associated adverse events or AEs).

For an individual patient with SCD, patterns in AEs, age at onset, frequency, duration, and response to therapy are not only prognostic but also mandate preemptive changes in health maintenance and monitoring strategies. While bone marrow transplantation is the only established cure for SCD, there are a growing number of preventive strategies that can improve morbidity, mortality, and quality of life.^4^ Evidence-based guidelines published by the National Institutes of Health (NIH) provide a reference for standard of care^6^^,^^7^ that has been widely accepted and applied for SCD. These guidelines, established by a team of SCD experts across the nation, were used to determine which data elements needed to be better captured at the bedside. The mapped data elements are then translated by a custom graphical user interface (GUI) into useful clinical knowledge which can be visualized to impact clinical care and research.

## MATERIALS AND METHODS

### Development and implementation of a SCD-specific data dictionary

The Nemours Sickle Cell Center of Biomedical Research Excellence Clinical Data Management team consists of a clinical applications analyst, bioinformatics experts, and SCD clinicians. The clinicians, a team of hematology physicians and nurse practitioners, worked with a clinical applications analyst to develop and implement a SCD Summary SmartForm (ESF) in EPIC. Content was based on the 2014 NIH SCD guidelines.^6^^,^^7^ Customizable EHR-based SmartForms (ESFs, structured data capture) provide the utility for capturing granular data at the bedside that could then be mapped with high quality to a data dictionary. Supplementary Table 1 lists the SmartData Elements contained in the ESF, and each data field is defined by a data type and a controlled set of values as defined in this data dictionary. The ESF has been made available in EPIC’s UserWeb.

**Table 1.** Adverse event severity grading scale for VOC and ACS

Grade	VOC	ACS
1	Home management	N/A
2	Clinic or ED management	Inpatient admission/intervention
3	Inpatient for analgesia ≤5 d	Intensive intervention such as ICU care, exchange transfusion
4	Inpatient for analgesia >5 d	Life threatening respiratory distress requiring intubation
5	N/A	Death

ACS, acute chest syndrome; ED, emergency department; VOC, vaso-occlusive crisis.

**Figure 1.**
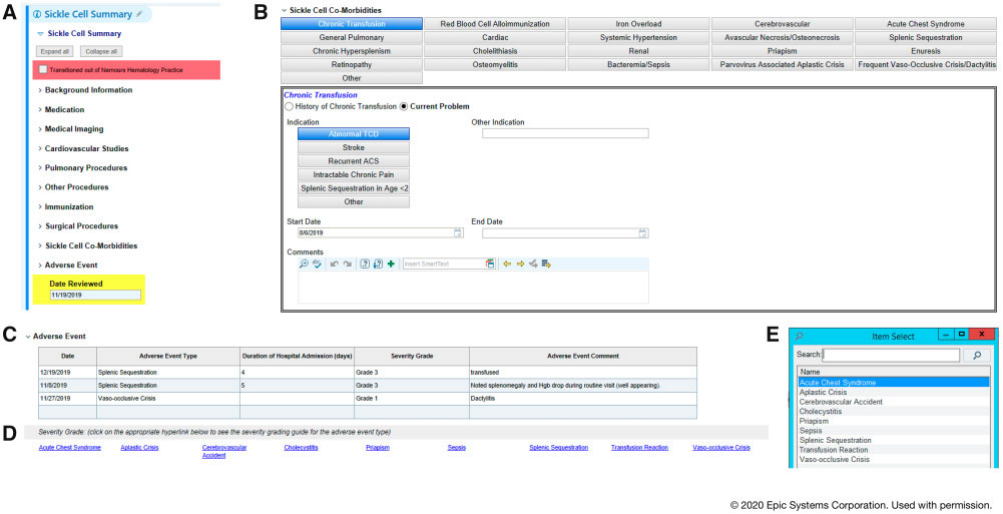
Sickle cell bedside data capture via an electronic healthcare record SmartForm. (A) The Sickle Cell SmartForm (ESF) is composed of 10 expandable sections. (B) This section of the Sickle Cell SmartForm allows providers to select from a menu of pertinent comorbidities that have previous or current impact on patient’s health. Once an item is selected, additional fields populate to allow for further description of the problem. This information, along with all data entered in the SmartForm, auto populate into a note by way of a dedicated SmartPhrase. The ability to view these details in a concise and organized format both guides and leverages clinical decision making at the bedside. For example, a provider may choose to transfuse red cells early in admission for a patient with a history of recurrent acute chest syndrome and current respiratory concerns. (C) The adverse event table allows providers to enter, grade, and track complications. (D) Hyperlinks are attached for easy viewing of grading scales. (E) Multiple complications are available for selection.

In addition to routine and baseline disease characteristics, the ESF contains 2 additional data types including (1) health maintenance information and (2) AE reporting. The ESF contains all aspects of SCD health surveillance including genotype, baseline lab values, hydroxyurea (HU) use, iron overload and chelation, imaging, cardiovascular and pulmonology studies, retinopathy screenings, neuropsychological testing, surgical procedures, pertinent immunizations, past and present comorbidities, and AEs (Figure 1A). A list of 20 SCD comorbidities available for selection and reporting is described in Figure 1B.

The ESF was built and validated via multiple iterative validation cycles, with clinicians returning to the informatics team with recommendations to enhance content and usability. Since the initial ESF was implemented, the SCD clinical team has continued to meet weekly with the informatics team to recommend changes to the ESF for continuous improvement of functionality. This process is critical as national disease guidelines are updated and new treatments modalities are introduced.

A severity grading scale for 9 acute SCD complications was developed, allowing providers to enter AE type and severity directly into the ESF (Figure 1C–E). The scales follow the approach used in the Common Terminology Criteria for Adverse Events published by the National Cancer Institute (https://ctep.cancer.gov) with AEs graded on a numeric scale of 1–5. Grading scales for ACS and VOC provided in Table 1.

### Custom application: Sickle Cell Knowledgebase

A custom GUI, referred to as the Sickle Cell Knowledgebase (SCK), was developed to visualize the mapped data elements. The SCK runs on a firewall-protected internal server using open-source technologies that utilize a RedHat Enterprise Linux v7.7, Apache v2.4.6, PostgreSQL v9.6, PHP v7.3.12, and Python v2.7.5 stack for development. This server is dedicated to the SCK application and consists of 8 CPUs, 16 GB of RAM, and has a response time of <0.1 s. The GUI builds on a Bootstrap v4.3.1 front-end component library and uses jQuery v3.3.1 and Google Charts for the tabular display of data as well as the graphical visualization of data (Table 2, Infrastructure).

**Table 2.** Overview of infrastructure, implementation metrics, and benefits

Infrastructure	Implementation metrics	Benefits
*Data*	*Provider data entry*	*Cohort identification*
Structured Electronic Healthcare Record Data	TCD = 99% Ophthalmology = 99% ACS G2–4 = 100% ACS G3–4 = 97% VOCs G3–4 = 100%	Patient-level dashboard improves review of health maintenance data
*Applications*	*Provider feedback*	
PostgreSQL Python PHP Google Charts	Benefits are incentive for changing bedside data entry	Utilized data standards and common data models for scalability

ACS, acute chest syndrome; TCD, Transcranial Doppler; VOC, vaso-occlusive crisis.

Initial efforts focused on patient demographics AE and health maintenance reporting. The architecture of the resulting tools was designed so that the client-side GUI sends calls to the server, where scripts query the database and return JSON formatted data to be rendered by the client-side libraries. Results are displayed as population-level data in interactive charts and visualizations, allowing clinicians to hover over elements for additional information or to click on elements to drill down to patient-level data. The linking of both tabular DataTables and Google Charts visualizations provides dual display of text and images. Charts and visualizations currently available include upcoming appointments and most recent visits to SCD clinic, demographics, health maintenance reports including vaccines and adherence to Transcranial Doppler (TCD) screening, HU dosing correlated with the occurrence and severity of AEs, and quantitation of iron load correlated with chelation dosing. Additional charts can be added to accommodate the needs of the specific user and patient population.

### Patient population

The study population includes all current patients with laboratory confirmed SCD actively followed at Nemours/Alfred I duPont Hospital for Children. Our population consists of 284 patients (53% female): 148 (52%) have hemoglobin (Hgb) SS, 95 (33%) Hgb SC, 25 (9%) Hgb S β^+^ Thalassemia, 9 (3%) Hgb S β ^0^ Thalassemia, and 7 (2%) with other genotypes. Twenty patients (7%) are younger than 24 months of age, 55 (19%) between 2 and 5 years, 97 (34%) between 6 and 11 years, 72 (25%) between 12 and 17 years, and 40 (14%) over 18 years of age. Data from all active patients were entered into the database at the outset of the project and new patients were added as they entered into the practice.

### Utilization assessment

#### Data entry

To demonstrate utility and scalability, we performed a 5-year retrospective chart review of 1374 inpatient encounters associated with ACS and VOC in 253 patients with SCD between 2010 and 2015. Retrospective VOC data were entered by a research coordinator and audited by a single experienced SCD provider. The following data points were reviewed for VOC: date of hospital admission, AE type, duration of admission, and severity grade. An audit plan was applied to conduct manual verification checks on a 10% random sample of retrospective patient encounters. The total error rate was calculated by dividing erroneous data points by total data points. The error rate was 3.5%. Based on published literature available for data audits in clinical research settings, a 5% error rate within electronic datasets is thought to be the gold standard.^8^ Ongoing data entry tracking active care is reviewed by all team members in weekly patient care meetings.

All ACS and other retrospective data were entered solely by an experienced SCD provider. Prospective data for all AEs from 2015 onward have been entered only by SCD providers, with ongoing internal quality checks performed each time a patient office visit is conducted and with each hospital discharge.

#### Utilization by SCD providers

To assess ESF utilization by SCD providers (3 nurse practitioners and 2 hematologists), the accuracy and completeness of the ESF records were evaluated. In the outpatient setting, entry of TCD exam and ophthalmology visit data at the time the SCD provider closed the visit encounter in EPIC were audited. TCD and ophthalmologic exams were chosen because these are representative standard of care screenings performed at yearly intervals. Data entered into the ESF were examined from all unique outpatient SCD clinic visit encounters for patients ≥24 months of age between April 1 and June 30, 2018 (*n* = 128) and compared to the corresponding EHR. In similar fashion, data from all inpatient hospital admissions were reviewed during the same 3-month period to assess entry of AE data from each hospitalization into the ESF. Completeness and accuracy were determined as the percentage of encounters in which queried data elements were present and correctly entered in the ESF.

##### Utilization by non-SCD providers

Using the associated SmartPhrase, a preconfigured text that allows the user to type a few characters that automatically expand into a longer block of text, the information in the ESF populates a provider’s progress note providing an organized and concise summary. Use of the ESF by emergency department (ED) and other non-SCD providers is desirable because it presents relevant data for each SCD patient in a standardized fashion, highlighting key markers of severity, important comorbidities and treatments critical to the acute care of these patients. To assess how the SCD Summary SmartForm was utilized by non-SCD providers in the ED and by admitting inpatient providers, all ED encounters (*n* = 194) and inpatient admissions (*n* = 136), occurring between January 1 and June 30, 2018, with a diagnosis related to SCD were identified using ICD10 codes. Utilization was calculated as the percentage of ED and initial inpatient history and physical notes which included the SCD SmartForm summary.

## RESULTS

### Implementation and utility of granular data entry system

**Figure 2.**
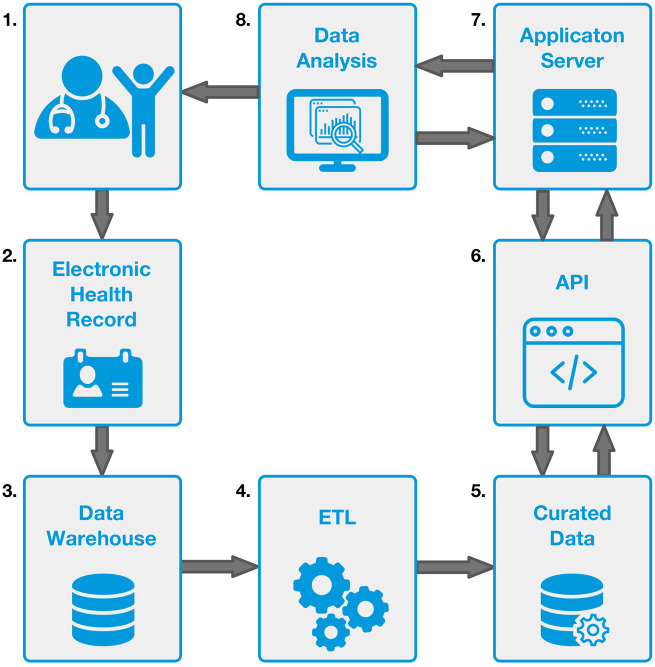
Dataflow. The learning health system data flow. (1) Clinician and patient encounters are documented with (2) patient data captured through traditional electronic healthcare record (EHR) sources along with committee defined Smart Form Data fields. (3) EHR data are processed nightly into our institution’s EPIC Clarity Data Warehouse. (4) Automated Extraction, Transformation, and Load (ETL) processes are run nightly using Pentaho Data Integration software (5) into a PostgreSQL Database. The data are formatted and optimized for auditing and reporting purposes. (6) A RESTful API built with the programing language Python and using the JSON format handles requests from the (7) LAMP stack application server. (8) Data visualization and reports are developed in collaboration with clinicians using Google Charts and DataTables.

A data dictionary with 175 SCD data elements classified as boolean, date, integer, float, lists, and text was created for SCD (Supplementary Table 1). Using a standardized clinical workflow, bedside data entry (Figure 2, panel 1) adhered to data standards and definitions outlined in the data dictionary. The data elements were extracted from the EHR, stored in the database (Figure 2, panels 2–5), and analyzed via a custom application (Figure 2, panels 6–8).

#### Implementation by SCD providers

To assess implementation of SmartForm data entry at the bedside, SCD providers were trained on use of the ESF. Analysis of data from SCD outpatient visit encounters over a 3-month period showed that SCD providers had entered dates for the last completed TCD exam and ophthalmology visit correctly 99% of the time (85/86 TCDs and 76/77 ophthalmology visits). Analysis of all inpatient admissions during the same period indicated that 100% (11/11) of all grade 2–4 ACS events, 97% (29/30) of all grade 3–4 VOCs, and 100% (7/7) of all grade 2–4 splenic sequestrations were entered into the AE section of the ESF. Together, these data indicate excellent compliance with timely completion of the ESF by SCD providers (Table 2, Implementation Metrics).

The knowledgebase application utilizes data entered at the bedside via structured data entry, termed SmartForms, and leverages data elements within the EHR. The application consists of a PostgreSQL database and uses Python, PHP, and JavaScript coding. Implementation metrics were analyzed regarding data entry for TCD, ophthalmology, ACS, and VOC grading, and providers were surveyed for feedback on the effectiveness of the system.

##### Utilization by ED and inpatient (non SCD) providers

Data collected over the first 6 months of 2018 demonstrated that the SmartPhrase summary was used to populate ED provider notes with information from the ESF in 50.5% (98/194) of SCD ED encounters. Over the same period, the SmartPhrase was used to populate the admission history and physical note in 69% (94/136) of SCD inpatient admissions. This likely represents an underestimation of the actual use of the ESF, since non-SCD providers often visualize the ESF data without entering the summary into their note.

Overall, the efficiency and clinical utility in the care of individual patients have led to a high degree of compliance with documentation requirements by SCD providers and voluntary use by inpatient and ED providers.

### Utilization of custom application (SCK) for patient-level data

**Figure 3.**
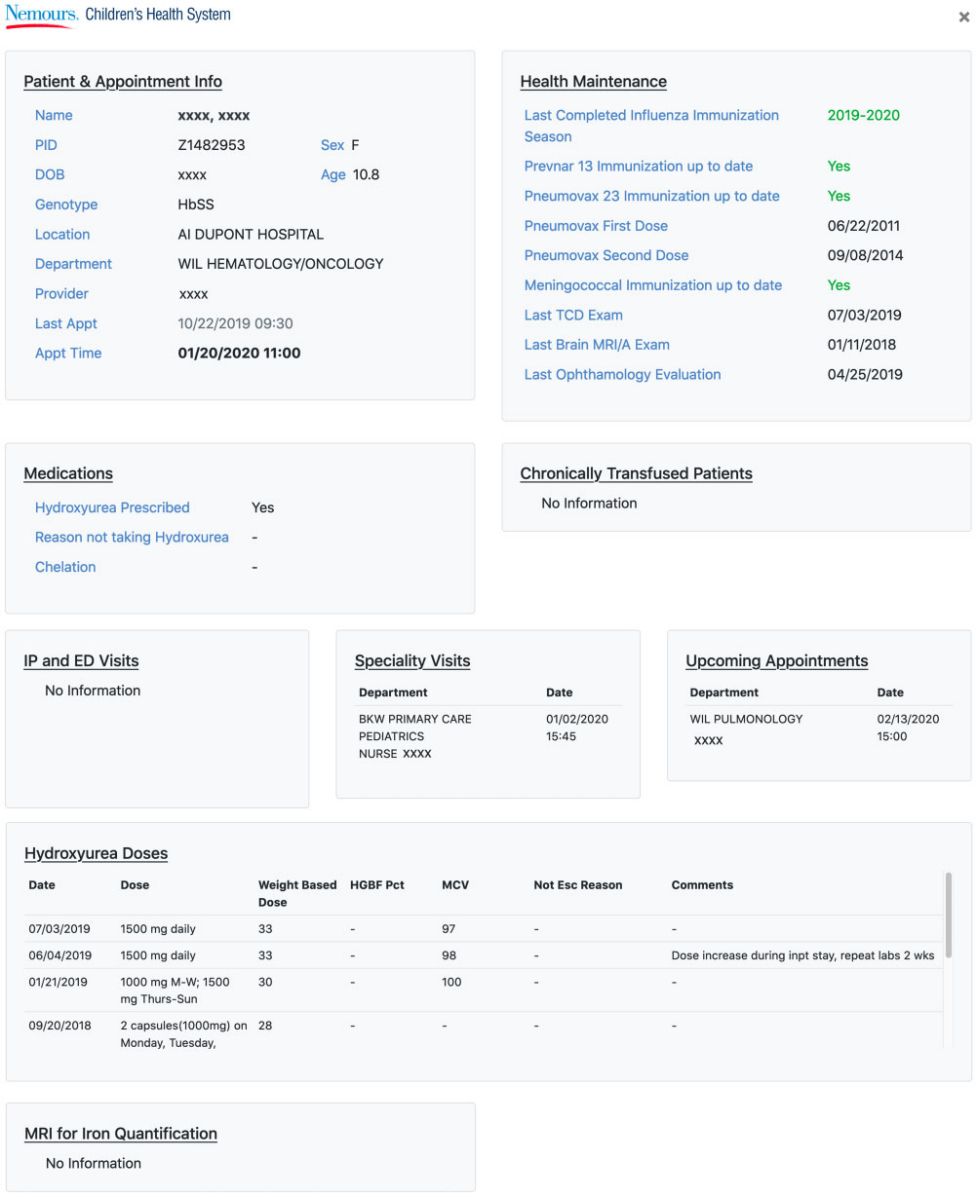
Automated chart review dashboard. This dashboard view allows for easy visualization of critical data needed for a provider to prepare for an upcoming sickle cell disease (SCD) clinic appointment. The information is broken down into major categories including basic demographics and appointment time, health maintenance adherence tracking, and information related to key SCD-modifying agents such as hydroxyurea (HU), upcoming specialty visits and appointments, and information on chelation relevant to chronically transfused patients.

A key incentive for physicians to entry granular data at the bedside is the enhanced visualization of relevant patient information that can typically take hours to compile using standard methods. The SCK provides easy access to individual patient reports that include baseline comorbidities, AEs, a health maintenance dashboard (Figure 3) with annotated delinquencies, and information on medication dosing and adherence. This dashboard decreases the amount of time required to review a patient chart prior to an outpatient visit, as these data elements are scattered throughout the EHR (Table 2, Benefits).

**Figure 4.**
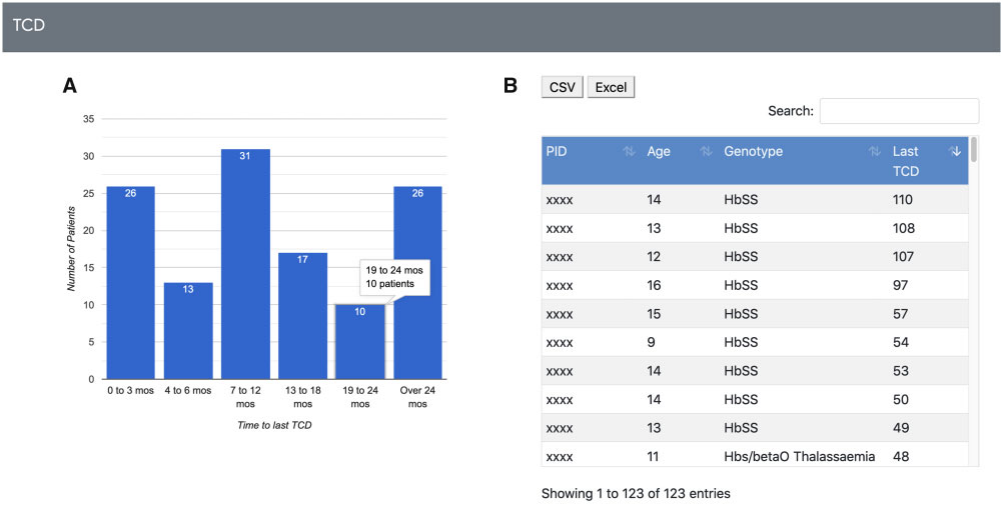
Sickle Cell Knowledgebase for analyzing key clinical metrics and health maintenance: focus on Transcranial Doppler (TCD). (A) Population-level report indicating of TCD monitoring timeliness. (B) Patient-level report indicating TCD adherence. Clicking on corresponding bar in (A) generates list shown in (B). First column lists patient identification (PID). Time is expressed as months from last TCD.

The SCK application is also utilized to monitor health maintenance screening for high risk patients. For example, TCD is recommended yearly for pediatric patients with SCD between 2 and 16 years of age (genotypes SS and S β ^0^ Thalassemia) and is a critical tool in the prevention of stroke.^9^ Using these reports, clinicians can efficiently identify patients in the appropriate age range with genotypes that require annual TCD monitoring and quickly capture the number (Figure 4A) and identity (Figure 4B) of patients overdue for surveillance.

### Utilization of custom application (SC knowledgebase) for population-level data

The SCK can generate population reports of AE frequency and severity, adherence to health maintenance recommendations, and comorbidities for individual patients in the context of a population of similarly managed patients (Table 2, Benefits).

Table 3 shows a snapshot of key population trends within our patient population identified through the LHS. Specifically, in population health maintenance, 39% of patients were overdue for TCD, and 57% of the SCD patients were overdue for annual retinopathy screening (Table 3a). The population comorbidities analysis yielded the following results: 4% patients with history of stroke, 47% patients with history of ACS, and 9% of patients on chronic transfusion therapy (Table 3b). For the population AEs between January 1, 2018 and July 1, 2019, the following trends were calculated: 31% of patients had a grade 3 and/or 4 VOC, 8% patients had ≥3 grade 3 or 4 VOCs, 2% patients had grade 3 and/or 4 ACS, and 0% patients had >1 grade 3 and/or 4 ACS (Table 3c).

**Table 3.** Identification of key population trends

**(a) Population health maintenance**	
39%	patients overdue for TCD US
57%	patients overdue for annual retinopathy screening
**(b) Population comorbidities**	
4%	patients with history of stroke
47%	of patients with history of ACS
9%	of patients on chronic transfusion therapy
**(c) Population averse events January 1, 2018 to July 1, 2019**	
31%	patients with grade 3 and 4 VOC
8%	patients with >3 grade 3/4 VOC
2%	patients with grade 3 and 4 ACS
0%	patients with >1 grade 3/4 ACS

Sickle Cell Knowledge Base allows for key trends within the patient population to be tracked by identifying percent/number of patients within specified groups who are (a) not up to date with recommended screenings, (b) suffered relevant comorbidities, or (c) who have suffered significant adverse events between January 1, 2018 and July 1, 2019. Data shown in table reflect patients with sickle cell disease at NAIDHC as of June 7, 2019. Within each of these populations, individual patients can be identified and targeted for appropriate clinical interventions or research studies. ACS, acute chest syndrome; TCD, Transcranial Doppler; VOC, vaso-occlusive crisis.

**Figure 5.**
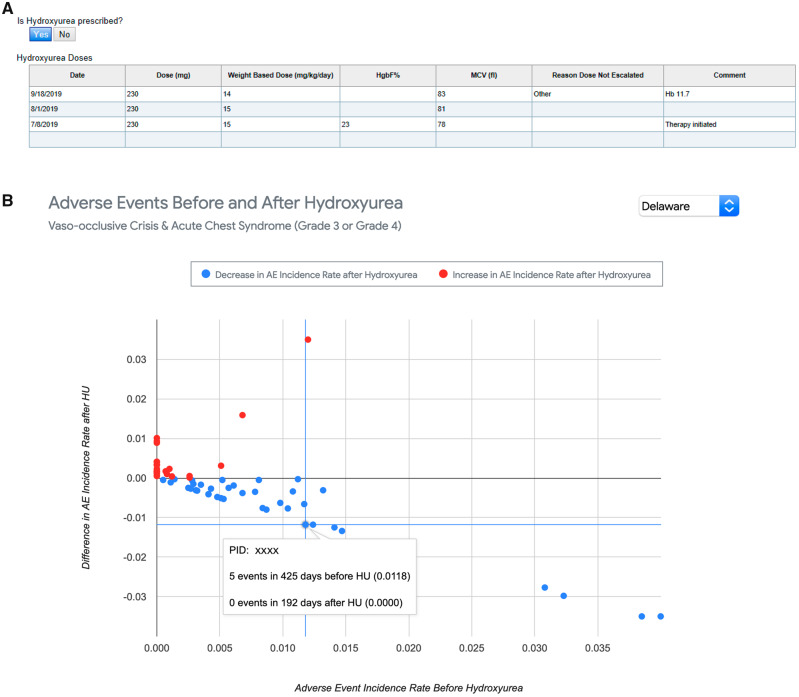
Sickle Cell Knowledgebase for hydroxyurea (HU) trend analysis. (A) HU is a very effective disease-modifying agent for patients with sickle cell disease (SCD). Close laboratory monitoring and frequent dose adjustments are required to ensure safe and effective therapy. (A) The HU dosing table exists in the ESF and allows prescribers to quickly view a patient’s dosing history, laboratory trend of efficacy, and any past toxicities to guide ongoing medication management. Providers enter new data into this table in real time. The same patient is depicted in (B inset), allowing for better visualization of the possible correlation between HU initiation and reduction in adverse event (AE) frequency. The patient depicted here had an excellent response to HU. (B) Population trends for AEs before and after initiation of HU therapy. Each dot represents a single patient plotted as AE incident ratio pre-HU therapy (*x*-axis) by AE incident delta post-HU therapy (*y*-axis). Clicking on each data point allows drill down to the corresponding patient’s AE data shown both prior to and after initiation of HU therapy.

**Figure 6.**
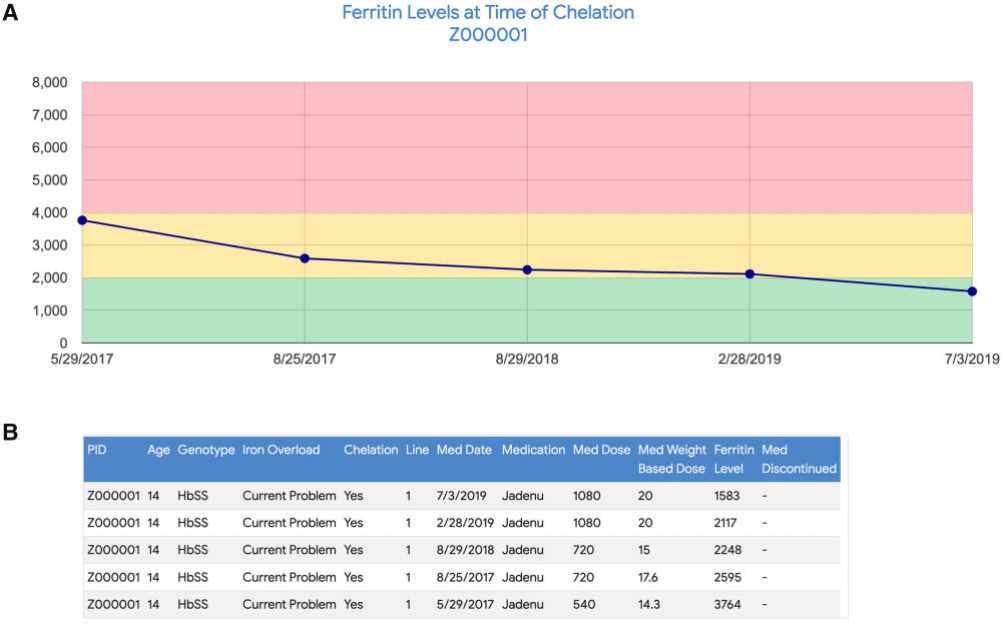
Sickle Cell Knowledgebase for iron chelation dosage and efficacy analysis. Jadenu is a medication used in patients requiring chronic transfusion therapy to treat iron overload. Efficacy over time can be tracked by trending serum ferritin levels. (A) A graphic representation of ferritin response over time from initiation of Jadenu and (B) dosage changes correlated with decreasing ferritin levels.

Figures 5 and 6 demonstrate the application for exploring specific outcomes in relation to selected therapies. HU is FDA approved for the prevention of SCD complications, works primarily through increasing fetal hemoglobin, and is highly safe and effective with appropriate dosing and laboratory monitoring.^10–13^ The ESF allows the clinician to track dose changes and reasons for dose modifications over time, adherence to required laboratory monitoring, and laboratory markers of efficacy (Figure 5A). Clinicians can view population trends for patients on HU as well as select and view an individual patient’s timeline of severe AEs in the context of HU therapy initiation and titration, providing an assessment of efficacy and dose optimization (Figure 5B). The patient depicted in Figure 5 had an excellent response to HU with a marked reduction in the incidence of AEs following the initiation of treatment (Figure 5B inset). Figure 6 provides another example of how the SCK can be used to track medication dosing and efficacy. Jadenu is a medication used to treat iron overload in patients requiring chronic transfusion therapy. Efficacy over time can be tracked by trending serum ferritin levels. Figure 6A shows a graphic representation of ferritin response from initiation of Jadenu, while Figure 6B shows specific dosage changes correlated with decreasing ferritin levels. In these examples, the ESF and SCK allowed for clear visualization of the correlation between treatment initiation and clinical response, providing a useful tool for compliance education.

To assess guideline adherence, the SCK was utilized to view the HU status of all patients in our practice with a genotype and age that would make them eligible for HU therapy, and who were not currently receiving chronic transfusions. We found that of 138 HU eligible patients, 68 were taking HU and 70 were not. Of the 70 not taking HU, it was possible to efficiently analyze the data and focus on patients to whom HU had not been offered and reach out to offer education about medication options.

The SCK can be used to identify eligible patients for clinical research studies and to collect data for those studies. For example, for an ongoing study which aims to improve implementation of HU therapy, the SCK was used to quickly identify patients currently on or offered HU and to assess healthcare utilization and SCD-related complications. To establish feasibility for a planned study, we were able to easily identify all subjects with eligible genotypes stratified by age and averaging ≥1 grade 3 or 4 VOCs per year (data not shown).

## DISCUSSION

By linking the efficient use of EHR tools to standardized data definitions with automated data abstraction, analytics, and visualization, a comprehensive LHS was realized. Our multidisciplinary team, utilizing a rich iterative feedback approach, developed an application that enables real-time analysis of critical aspects of SCD clinical care. The system improves the efficiency of data entry, provides a single point of entry for a complete patient-level data summary, and facilitates patient care.

Clinicians have experienced an improvement in documentation efficiency. Clinical data entered into the ESF need only be entered once, rather than at the time of each patient encounter. Relevant, accurate data can then be easily pulled into an encounter note using an EPIC SmartPhrase. This process reduces the time required to create encounter progress notes. Thus far, we have demonstrated a very high level of accuracy and completeness of data entered into the ESFs. We will continue to monitor data quality at regular intervals, at least annually, to ensure that these high levels are consistently maintained.

In addition to formal assessments, the quality of data is assessed and updated on a continuing basis by the SCD providers at the time of each clinical visit and hospital discharge. The ESFs and SCK are utilized at a weekly SCD team meeting in which data from all patients coming to clinic in the upcoming week are reviewed. Maintaining up to date and high quality ESF data is considered an integral role of each SCD provider and is included as part of their annual performance assessment. Finally, it should be noted that *only* SCD providers can enter data into the ESFs, further ensuring accuracy of data.

Patient outcomes and adherence to recommended care will be tracked over time to measure efficacy.

This system can be used for patient education and shared decision making. For instance, current NIH guidelines recommend offering HU to all children with Hgb SS or Hgb Sβ^0^ Thalassemia starting at 9 months of age. When these guidelines were released in 2014, this represented a distinct change from the previous standard of care and since then, our SCD clinical team has strived to increase uptake of HU. The SCK enables patients to review their own data in relation to a population and also their own clinical course with their provider to clarify recommendations.

AEs and laboratory data can be tracked in the context of the institution of new therapies and changes in medication doses to assess efficacy. Outcomes can also be assessed on a population basis. These capabilities become increasingly important as new therapies are introduced. Several new disease-modifying agents have recently been approved by the FDA for use in SCD with limited data on how best to utilize and combine these agents with existing therapies. Clinicians will need to carefully monitor positive responses and AEs that may occur as new drugs and combinations come into use.

The data entry system enables the collection of records for a lifetime of care and also serves as a comprehensive and succinct summary, which can be utilized when patients transfer their care to a new institution or graduate to adult care providers. Previously, the preparation of a comprehensive summary for a complex patient could take a provider as much as a full day. Using the ESF, a summary can be created in just a few minutes. The ESF summary is also highly utilized by our ED, consultant, and inpatient care clinicians to guide medical decision making on a much more personalized basis. The ability to easily access critical patient data entered and curated by content experts greatly facilitates accurate communication between providers to ensure high quality and safe care.^14^

In addition to its clinical utility, the granular data entry has greatly enhanced the ability to access patient data for research. Granular data elements mapped to the data dictionary can be easily parsed and searched to identify patient cohorts eligible for research studies and to generate hypotheses based on real-world data. Common data elements that were previously extremely time consuming to collect and verify, such as frequency and severity of VOC in study participants, are now accurately recorded and easily accessible. By implementing this type of bedside data-entry-based common terminology, descriptors, and data models, multiple healthcare systems can link data to facilitate collaborative research. An added benefit is that data in the SCK can be visualized in a deidentified format for use in research.

### Limitations

While we have demonstrated the usability and benefits derived from our system within the relatively small patient population at our institution, we did not set out to prove that it would improve outcomes. Doing this will require additional longitudinal follow-up and a larger patient population. Our intention was to create tools and a model for SCD that can be adapted and tested by other healthcare systems and across many chronic illnesses. Our patient population was small enough that a thorough audit of retrospective data could be done by a single expert with dedicated time. However, we acknowledge that use of a single reviewer could have introduced some bias. While the time saved by providers utilizing our system is clear to all who use it, it was not feasible to provide a quantitative measure of time saved. Thus, the discussion of efficiency is based on subjective observations.

### Future directions

Future work will focus on communication between patient portals and the EHR to improve both adherence to care recommendations and communication outside the network of regional care providers. Additionally, our group is currently developing similar ESFs for use in pediatric acute leukemia and diabetes care.

## CONCLUSION

Data dictionaries, standardized bedside data entry, and a multidisciplinary team are essential for developing an effective and scalable environment capable of realizing a SCD LHS. While we have chosen to focus on SCD, this strategy for implementing an LHS has broad applicability to many other complex chronic disease models such as asthma, cancer, diabetes, or heart disease.

## SIGNIFICANCE

The successful creation of an LHS will be dependent on healthcare institutions adopting standardized processes for data collection, storage, transfer, analysis, and destruction. This manuscript is timely and relevant as it provides steps necessary to create a system capable of realizing precision medicine.

## FUNDING

Research reported in this publication was supported by the National Institute of General Medical Sciences of the National Institutes of Health under award number P20GM109021 and an Institutional Development Award (IDeA) from the National Institute of General Medical Sciences of the National Institutes of Health under grant number U54-GM104941. Development of the Nemours PEDSnet data resource was supported by Patient-Centered Outcomes Research Institute (PCORI) award number 1306-01556.

## AUTHOR CONTRIBUTIONS

RM, EC, JW, RG, EAK, and RV developed and implemented the SCD Summary SmartForm in EPIC. SJ-A abstracted historical data into the system. ELC, DE, and JCM developed the Sickle Cell Knowledgebase. SLV provided expert assistance in design of both the ESF and SCK. All authors contributed to the writing of the manuscript.

## CONFLICT OF INTEREST STATEMENT

None declared.

## Supplementary Material

ooaa024_Supplementary_Data
