# Supplementary material for: Implementation of a learning healthcare system for sickle cell disease
Source: JAMIA Open. 2020 Oct 23;3(3):349–59. doi: 10.1093/jamiaopen/ooaa024 (PMC7660956; doi:10.1093/jamiaopen/ooaa024)
Supplement: ooaa024_Supplementary_Data [file ooaa024_supplementary_data.pdf]

Supplemental Table 1 – List of Smart Data Elements contained in the ESF and their SmartData type

Group	Field	Condition	Type	Src *
**Background Information**	Sickle Cell Genotype Specification		SELECT<br>[HgbSS,<br>HgbSC,<br>Hbs/beta + Thalassemia,<br>Hbs/betaO Thalassemia,<br>Other]	A
	Sickle Cell Genotype Comment		Text	A
	Sickle Cell Baseline Hemoglobin Range		Text	A
	Sickle Cell Baseline Reticulocyte		Text	A
	Sickle Cell Baseline WBC		Text	A
	Transitioned Out Of Hematology Practice		Boolean	A
	Death		Date	B
	Sickle Cell Visit Information Date Reviewed		Date	A
**Medication**	Sickle Cell Hydroxyurea Treatment *1		Boolean	A
	Sickle Cell Hydroxyurea Treatment Date	*if *1 = YES*	Date	A
	Sickle Cell Hydroxyurea Dose (mg)	*if *1 = YES*	Text	A
	Sickle Cell Hydroxyurea Weight Based Dose (mg/kg/day)	*if *1 = YES*	Integer	A
	Fetal Hemoglobin Level At Hydroxyurea Treatment (%)	*if *1 = YES*	Float (4,1)	A
	MCV Level At Hydroxyurea Treatment (fl)	*if *1 = YES*	Float (5,1)	A
	Reason Dose Not Escalated	*if *1 = YES*	SELECT<br>[At intended dose,<br>Neutropenia,<br>Thrombocytopenia,<br>GI distress,<br>Patient/Family reluctance,<br>Other]	A
	Sickle Cell Hydroxyurea Treatment Comments	*if *1 = YES*	Text	A
	Reason Not Taking Hydroxyurea *2	*If *1 = NO*	SELECT<br>[Not indicated,<br>Patient refused,<br>On chronic transfusion,<br>Prior adverse reaction,<br>Other]	A
	Hydroxyurea Additional Comments	*If *2 = NO*	Text	A
	Sickle Cell Pain Plan Contract Signed		Date	A
	Last Opioid Inform Consent Signed		Timestamp	A
	Last TCD Exam		Date	A
	Last TCD Exam Results		SELECT [Normal, Abnormal, Conditional]	A
	TCD Comments		Text	A
	Last Brain MRI/A Exam		Date	A
	Last Brain MRI/A Exam Results		SELECT [Normal, Abnormal, Unchanged]	A
	Brain MRI/A Comments		Text	A
	MRI For Iron Quantification Date		Date	A
	MRI For Iron Quantification Results		SELECT [Normal, Abnormal]	A
	Cardiac Iron Ferriscan Value		Text	A
	Cardiac Iron Ge Level		Text	A
	Liver Iron Ferriscan Value		Text	A
	Liver Iron Ge Value		Text	A
	Last Abdominal Ultrasound		Date	A

Supplemental Table 1 – List of Smart Data Elements contained in the ESF and their SmartData type

Group	Field	Condition	Type	Src *
Imaging	Last Abdominal Ultrasound Results		SELECT [Normal, Abnormal]	A
	Abdominal Ultrasound Comments		Text	A
	Last Renal Ultrasound		Date	A
	Last Renal Ultrasound Results		SELECT [Normal, Abnormal]	A
	Renal Ultrasound Comments		Text	A
	Last MRCP		Date	A
	Last MRCP Results		SELECT [Normal, Abnormal]	A
	MRCP Comments		Text	A
	Last Shoulder Xray		Date	A
	Last Shoulder XRay Results		SELECT [Normal, Abnormal]	A
	Last Shoulder Xray Comments		Text	A
	Last Shoulder MRI		Date	A
	Last Shoulder MRI Results		SELECT [Normal, Abnormal]	A
	Last Shoulder MRI Comments		Text	A
	Last Hip Xray		Date	A
	Last Hip Xray Results		SELECT [Normal, Abnormal]	A
	Last Hip Xray Comments		Text	A
	Last Hip MRI		Date	A
	Last Hip MRI Results		SELECT [Normal, Abnormal]	A
	Last Hip MRI Comments		Text	A
Cardiovascular Procedures	Last Echo Exam		Date	A
	Last Echo Exam Results		SELECT [Normal, Abnormal]	A
	Last Echo Exam Comments		Text	A
	Last EKG Exam		Date	A
	Last EKG Result		SELECT [Normal, Abnormal]	A
	Last EKG Result Comments		Text	A
	Other Cardiac Test		Text	A
	Other Cardiac Test Date		Date	A
	Other Cardiac Test Comments		Text	A
Pulmonary Procedures	Last PFT's Exam		Date	A
	PFT's Results		SELECT [Normal, Abnormal]	A
	PFT's Results Comments		Text	A
	Last Spirometry Exam		Date	A
	Last Spirometry Exam Results		SELECT [Normal, Abnormal]	A
	Last Spirometry Exam Comments		Text	A
	Last Sleep Study Exam		Date	A
	Sleep Study Results		SELECT [Normal, Abnormal]	A
	Sleep Study Results Comments		Text	A
	Respiratory Support		SELECT [Overnight Oxygen, CPaP]	A
Other Procedures	Last Ophthalmology Examination		Date	A
	Ophthalmology Evaluation Results		SELECT [Retinopathy, No Retinopathy]	A
	Ophthalmology Evaluation Comments		Text	A
	Last Audiogram		Date	A
	Audiogram Results		SELECT [Normal, Abnormal]	A
	Audiogram Results Comments		Text	A
	Neuropsych Testing		Text	A
	Neuropsych Testing Date		Date	A
	Neuropsych Testing Result		Text	A

Supplemental Table 1 – List of Smart Data Elements contained in the ESF and their SmartData type

Group	Field	Condition	Type	Src *
**Immunizations**	Last Completed Influenza Immunization Season		SELECT [2018-2019, 2017-2018, 2016-2017, 2015-2016, 2014-2015, Before 2014-2015, None, Not Applicable]	A
	Meningococcal Immunization Up To Date		SELECT [Yes, No, Unknown]	A
	Pneumovax 23 Immunization Up To Date		SELECT [Yes, No, Unknown]	A
	Prevnar 13 Immunization Up To Date		SELECT [Yes, No, Unknown]	A
	Additional Immunization Comments		Text	A
**Surgical Procedures**	CVL Placement number		Integer	A
	CVL Type		SELECT [Port, PICC, Tunneled, Non-Tunneled, Apheresis, Arterial line]	A
	Central Venous Line Lumen		Integer	A
	CVL Placement Date		Date	A
	CVL Removal Date		Date	A
	CVL Removal Reason		SELECT [Routine, Therapy Complete, Temporary Line, Infection, Functional Issues, Thrombus]	A
	History Of Cerebrovascular Surgery		Boolean	A
	Date Of Cerebrovascular Surgery		Date	A
	History Of Cholecystectomy		Boolean	A
	Date Of Cholecystectomy		Date	A
	History Of ERCP		Boolean	A
	Date Of ERCP		Date	A
	History Of Joint Surgery		Boolean	A
	Date Of Joint Surgery		Date	A
	History Of Splenectomy		Boolean	A
	Date Of Splenectomy		Date	A
	History Of T & A		Boolean	A
	Date Of T & A		Date	A
	Other Surgery Type		Text	A

Supplemental Table 1 – List of Smart Data Elements contained in the ESF and their SmartData type

Group	Field	Condition	Type	Src *
	Sickle Cell Co-Morbidities *3		SELECT<br>[Acute Chest Syndrome,<br>Avascular Necrosis/Osteonecrosis,<br>Bacteremia/Sepsis,<br>Cardiac,<br>Cerebrovascular,<br>Cholelithiasis,<br>Chronic Hypersplenism,<br>Chronic Transfusion,<br>Enuresis,<br>Frequent Vaso-Occlusive Crisis/Dactylitis,<br>General Pulmonary,<br>Iron Overload,<br>Osteomyelitis,<br>Parvovirus Associated Aplastic Crisis,<br>Priapism,<br>Red Blood Cell Alloimmunization,<br>Renal,<br>Retinopathy,<br>Splenic Sequestration,<br>Systemic Hypertension,<br>Other]	A
	Acute Chest Syndrome	*if *3 = Acute Chest Syndrome*	SELECT [Current Problem, History of Acute Chest Syndrome]	A
	Acute Chest Syndrome Comments	*if *3 = Acute Chest Syndrome*	Text	A
	Avascular Necrosis/Osteonecrosis	*if *3 = Avascular Necrosis/Osteonecrosis*	SELECT [Current Problem, History of Avascular Necrosis/Osteonecrosis]	A
	Sickle Cell Avascular Necrosis Co-Morbidity Affected Limb	*if *3 = Avascular Necrosis/Osteonecrosis*	Select [Shoulder, Hip, Knee, Other]	A
	Avascular Necrosis/Osteonecrosis Comments	*if *3 = Avascular Necrosis/Osteonecrosis*	Text	A
	Bacteremia/Sepsis	*if *3 = Bacteremia/Sepsis*	SELECT [Current Problem, History of Bacteremia/Sepsis]	A
	Bacteremia/Sepsis Comments	*if *3 = Bacteremia/Sepsis*	Text	A
	Sickle Cell Cardiac History	*if *3 = Cardiac*	SELECT [Current Problem, History of Cardiac Comorbidity]	A
	Sickle Cell Cardiac Co-Morbidities	*if *3 = Cardiac*	SELECT [Pulmonary Hypertension, Cardiomyopathy]	A
	Sickle Cell Cardiac Comments	*if *3 = Cardiac*	Text	A

Supplemental Table 1 – List of Smart Data Elements contained in the ESF and their SmartData type

Group	Field	Condition	Type	Src *
Co-morbidities	Sickle Cell Cerebrovascular History	*if *3 = Cerebrovascular*	SELECT [History of stroke or TIA with no imaging or neurologic findings, Abnormal TCD/no stroke, Conditional TCD/no stroke, Abnormal MRI and/or TCD, With a history of stroke, no residual deficits, Abnormal MRI and/or TCD with a history of stroke and residual neurologic deficits, Abnormal MRI and/or TCD with a history of stroke, cerebral artery occlusion, Moya moya (with or without residual deficits)]	A
	Cerebrovascular Comments	*if *3 = Cerebrovascular*	Text	A
	Cholelithiasis	*if *3 = Cholelithiasis*	SELECT [Current Problem, History of Cholelithiasis]	A
	Sickle Cell Gallbladder Issues	*if *3 = Cholelithiasis*	SELECT [Stones, History of Cholecystitis, Sludge, Dilation of Bile Duct]	A
	Cholelithiasis Comments	*if *3 = Cholelithiasis*	Text	A
	Chronic Hypersplenism	*if *3 = Chronic Hypersplenism*	SELECT [Current Problem, History of Chronic Hypersplenism]	A
	Baseline Spleen Size	*if *3 = Chronic Hypersplenism*	Float (4,1)	A
	Spleen Guard	*if *3 = Chronic Hypersplenism*	Boolean	A
	Hypersplenism Comments	*if *3 = Chronic Hypersplenism*	Text	A
	Chronic Transfusion	*if *3 = Chronic Transfusion*	SELECT [Current Problem, History of Chronic Transfusion]	A
	Transfusion Indication	*if *3 = Chronic Transfusion*	SELECT [Abnormal TCD, Stroke, Recurrent ACS, Intractable Chronic Pain, Splenic Sequestration in Age < 2, Other]	A
	Transfusion Start Date	*if *3 = Chronic Transfusion*	Date	A
	Transfusion End Date	*if *3 = Chronic Transfusion*	Date	A
	Transfusion Comments	*if *3 = Chronic Transfusion*	Text	A
	Sickle Cell Enuresis History	*if *3 = Enuresis*	SELECT [Current Problem, History of Enuresis]	A
	Sickle Cell Enuresis Comments	*if *3 = Enuresis*	Text	A
	Frequent Vaso-Occlusive Crisis/Dactylitis	*if *3 = Frequent Vaso-Occlusive Crisis/Dactylitis*	SELECT [Current Problem, History of Frequent Vaso-Occlusive Crisis/Dactylitis]	A

Supplemental Table 1 – List of Smart Data Elements contained in the ESF and their SmartData type

Group	Field	Condition	Type	Src *
	Frequent Vaso-Occlusive Crisis/Dactylitis Comments	*if *3 = Frequent Vaso-Occlusive Crisis/Dactylitis*	Text	A
	Frequent Vaso-Occlusive Crisis/Dactylitis History	*if *3 = Frequent Vaso-Occlusive Crisis/Dactylitis*	SELECT [Frequent VOC, Chronic Pain, Dactylitis]	A
	Sickle Cell General Pulmonary History	*if *3 = General Pulmonary*	SELECT [Current Problem, History of General Pulmonary Comorbidity]	A
	Pulmonary Comorbidities	*if *3 = General Pulmonary*	SELECT [Asthma, Obstructive Sleep Apnea, Pulmonary Hypertension]	A
	Sickle Cell Pulmonary Comments	*if *3 = General Pulmonary*	Text	A
	Iron Overload	*if *3 = Iron Overload*	SELECT [Current Problem, History of Iron Overload]	A
	Chelation	*if *3 = Iron Overload*	Boolean	A
	Sickle Cell Chelation Medication	*if *3 = Iron Overload*	SELECT [Exjade, Jadenu, Desferal]	A
	Sickle Cell Chelation Med Date	*if *3 = Iron Overload*	Date	A
	Sickle Cell Chelation Med Dc Date	*if *3 = Iron Overload*	Date	A
	Sickle Cell Chelation Med Dose (mg)	*if *3 = Iron Overload*	Text	A
	Sickle Cell Chelation Med Weight Based Dose (mg/Kg)	*if *3 = Iron Overload*	Float (5,2)	A
	Ferritin Value At Chelation Treatment	*if *3 = Iron Overload*	Text	A
	Sickle Cell Chelation Meds Comments	*if *3 = Iron Overload*	Text	A
	Iron Overload Comments	*if *3 = Iron Overload*	Text	A
	Sickle Cell Osteomyelitis History	*if *3 = Osteomyelitis*	SELECT [Current Problem, History of Osteomyelitis]	A
	Sickle Cell Osteomyelitis Comments	*if *3 = Osteomyelitis*	Text	A
	Parvovirus Associated Aplastic Crisis	*if *3 = Parvovirus Associated Aplastic Crisis*	SELECT [Current Problem, History of Parvovirus Associated Aplastic Crisis]	A
	Sickle Cell Parvovirus Co-Morbidity IgG Positive?	*if *3 = Parvovirus Associated Aplastic Crisis*	Boolean	A
	Parvovirus Associated Aplastic Crisis Comments	*if *3 = Parvovirus Associated Aplastic Crisis*	Text	A
	Priapism History	*if *3 = Priapism*	SELECT [Current Problem, History of Priapism]	A
	Priapism Comments	*if *3 = Priapism*	Text	A
	Red Blood Cell Alloimmunization	*if *3 = Red Blood Cell Alloimmunization*	SELECT [Current Problem, History of Red Blood Cell Alloimmunization]	A
	Red Blood Cell Alloimmunization Comments	*if *3 = Red Blood Cell Alloimmunization*	Text	A
	Sickle Cell Renal History	*if *3 = Renal*	SELECT [Current Problem, History of Renal Comorbidity]	A
	Sickle Cell Renal Issues	*if *3 = Renal*	SELECT<br>[Proteinuria,<br>Recurrent UTI,<br>Papillary Necrosis,<br>Abnormal 24 Hour ABPM Study]	A
	Sickle Cell Renal Comments	*if *3 = Renal*	Text	A
	Sickle Cell Retinopathy History	*if *3 = Retinopathy*	SELECT [Current Problem, History of Retinopathy]	A
	Sickle Cell Retinopathy Comments	*if *3 = Retinopathy*	Text	A

Supplemental Table 1 – List of Smart Data Elements contained in the ESF and their SmartData type

Group	Field	Condition	Type	Src *
	Splenic Sequestration	*if *3 = Splenic Sequestration*	SELECT [Current Problem, History of Splenic Sequestration]	A
	Splenic Sequestration Comments	*if *3 = Splenic Sequestration*	Text	A
	Systemic Hypertension	*if *3 = Systemic Hypertension*	SELECT [Current Problem, History of Systemic Hypertension]	A
	Systemic Hypertension Comments	*if *3 = Systemic Hypertension*	Text	A
	Other Sickle Cell Co-morbidity	*if *3 = Other*	Text	A
**Adverse Events**	Sickle Cell Adverse Event Type		SELECT [Vaso-occlusive Crisis, Acute Chest Syndrome, Splenic Sequestration, Aplastic Crisis, Sepsis, Cerebrovascular Accident, Cholecystitis, Priapism]	A
	Sickle Cell Adverse Event Date		Date	A
	Sickle Cell Adverse Event Severity Grading		SELECT [Grade 1, Grade 2, Grade 3, Grade 4, Grade 5]	A
	Sickle Cell Adverse Event Duration Of Hospital Admission (Days)		Integer	A
	Sickle Cell Adverse Event Comments		Text	A
**Encounters**	Date of last Hemoglobin Electrophoresis OR Cascade Electrophoresis		Date	B
	Date of last resulted CBC/diff		Date	B
	Last completed hematology/oncology office visit		Date	B
	Last ED encounter		Date	B
	Last inpatient hospital admission		Date	B
	Last Nephrology visit		Date	B
	Last Pulmonology visit		Date	B

* Data Source: A = EPIC Smartform Data Element, B = PEDSnet
